# Supplementary figures and images for: Transcriptomic analysis reveals cloquintocet-mexyl-inducible genes in hexaploid wheat (Triticum aestivum L.)
Source: PLoS One. 2025 Feb 18;20(2):e0319151. doi: 10.1371/journal.pone.0319151 (PMC11835315; doi:10.1371/journal.pone.0319151)

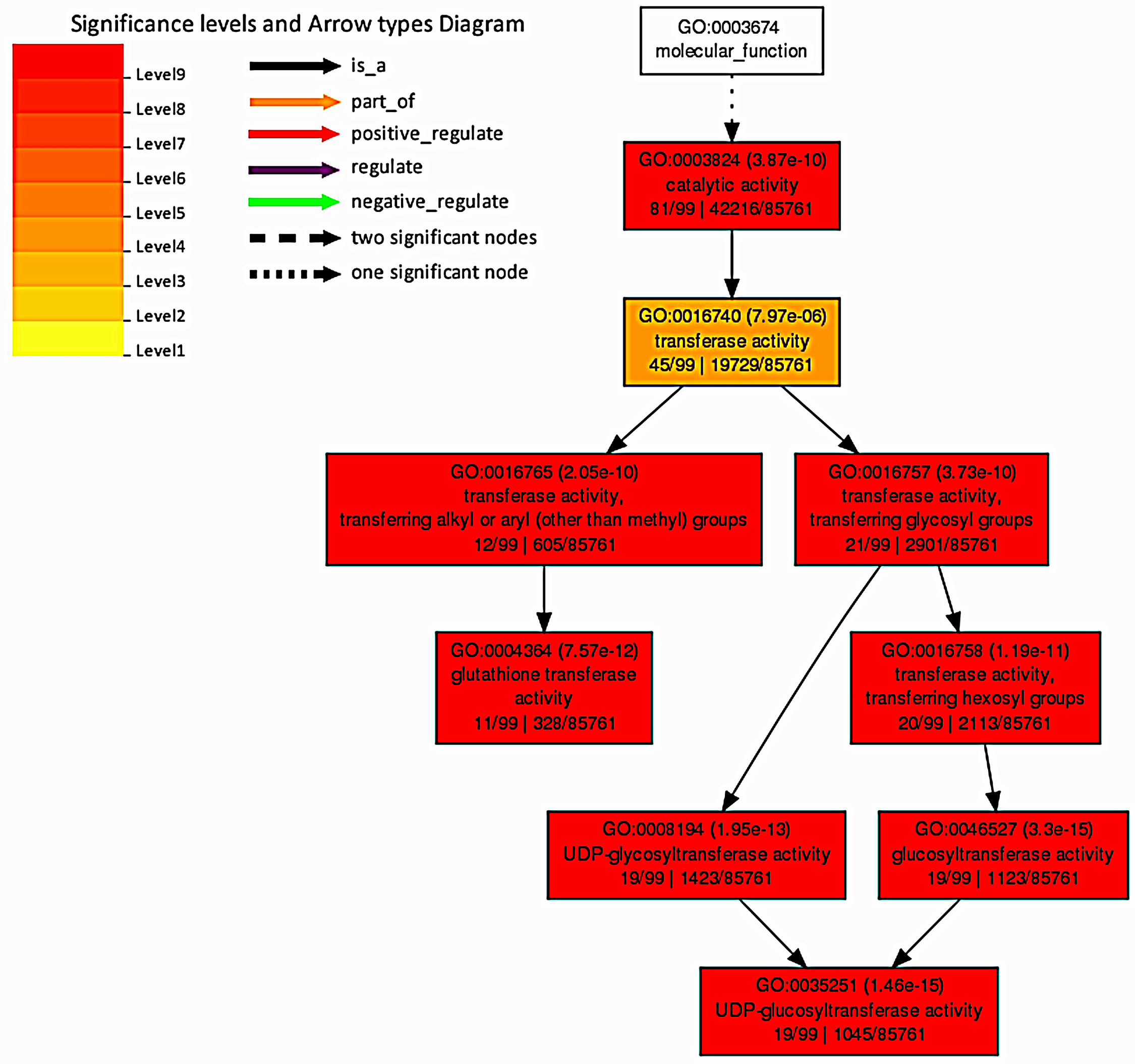

Supplement: S1 Fig — The color of the box indicates the significance level of the false discovery rate (reported in parentheses), with the yellow indicating relatively low significance and the gradation intensifies towards red to indicate higher significance. At the bottom of each significant box, the first fraction represents the number of significant differentially expressed genes assigned the specified GO term (out of 99), and the second fraction indicates the number of genes in the Triticum aestivum L. reference background with the same GO annotation. (TIF) [file pone.0319151.s001.tif]

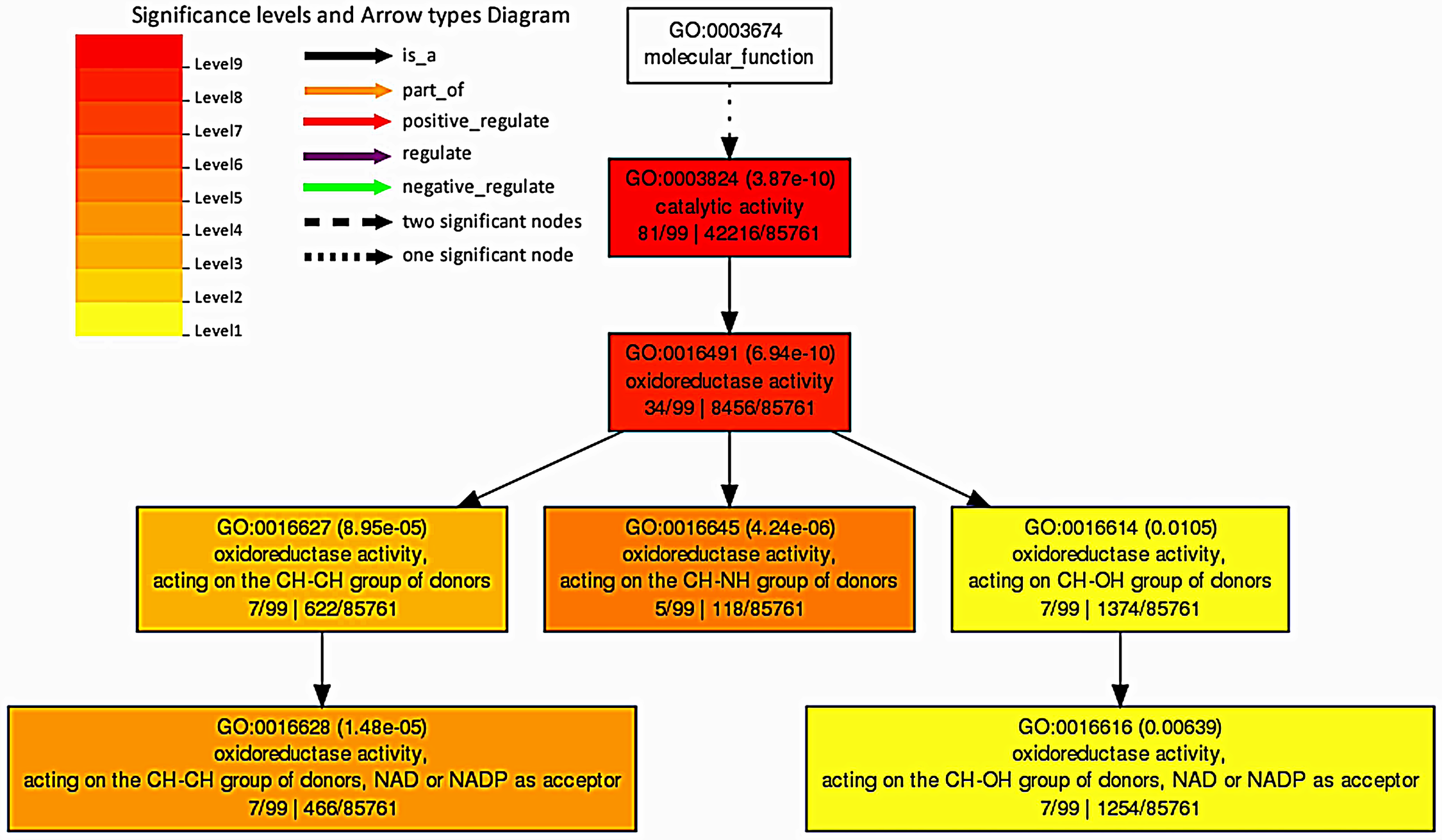

Supplement: S2 Fig — The color of the box indicates the significance level of the false discovery rate (reported in parentheses), with the yellow indicating relatively low significance and the gradation intensifies towards red to indicate higher significance. At the bottom of each significant box, the first fraction represents the number of significant differentially expressed genes assigned the specified GO term (out of 99), and the second fraction indicates the number of genes in the Triticum aestivum L. reference background with the same GO annotation. (TIF) [file pone.0319151.s002.tif]

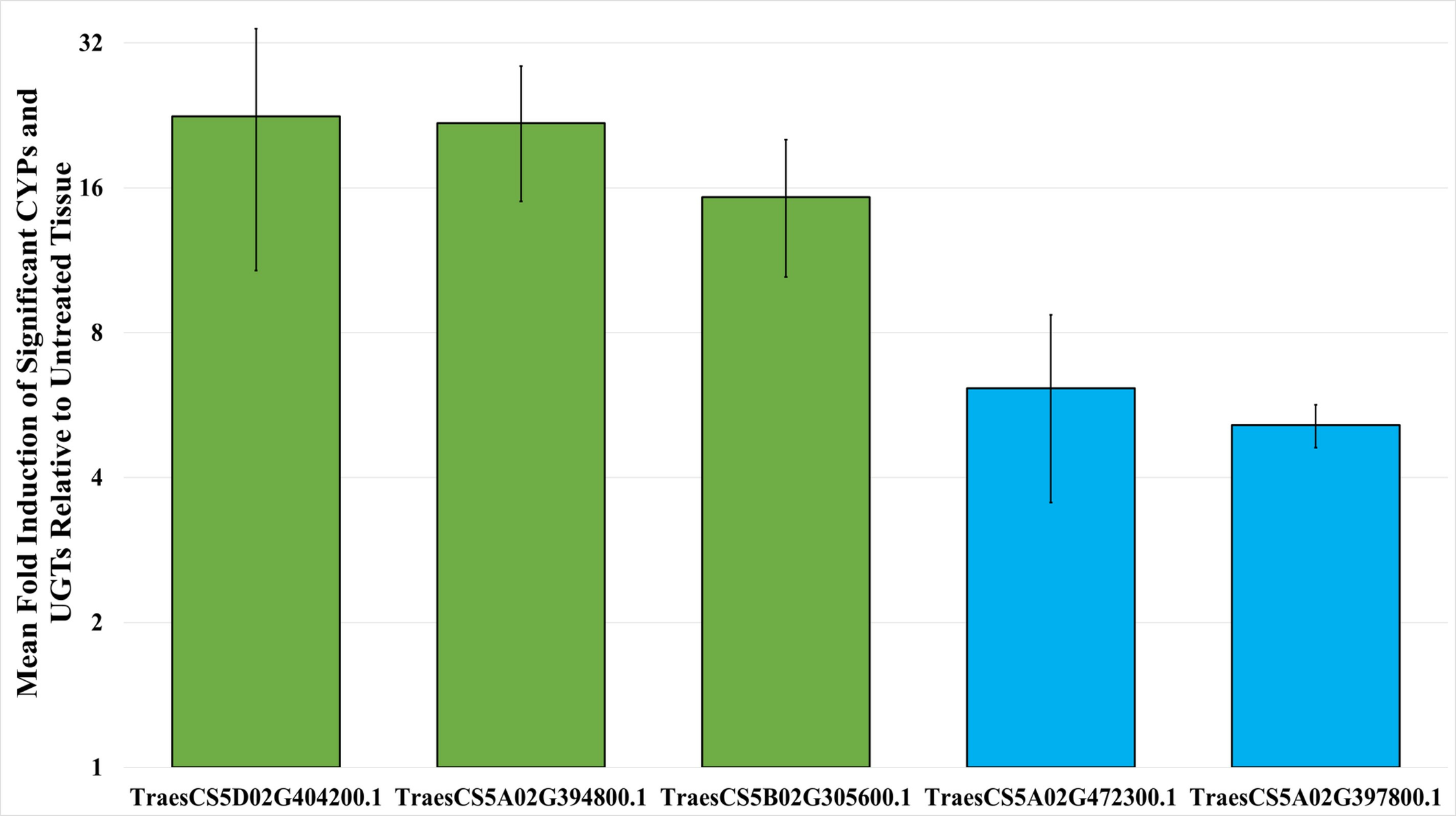

Supplement: S3 Fig — Green bars represent UGTs and blue bars represent CYPs. Genes were induced by 15 g a.i. ha-1 of cloquintocet-mexyl relative to untreated controls. Error bars indicate standard error of the mean. (TIF) [file pone.0319151.s003.tif]

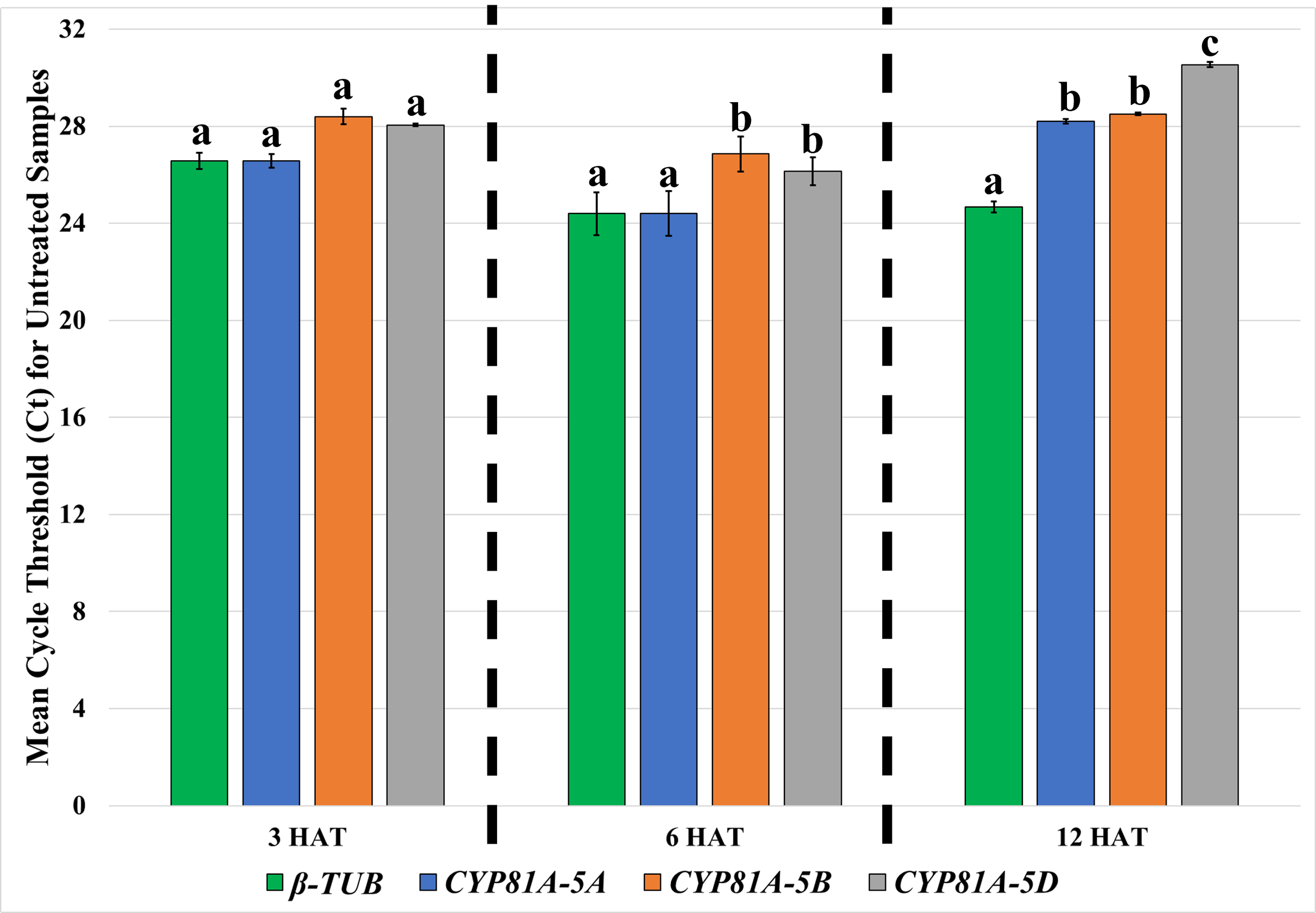

Supplement: S4 Fig — The UT treatment consisted of 0.1% nonionic surfactant, which is an adjuvant that was included in all other treatment utilized for this experiment. Within each timepoint, means that share the same letter are not significantly different (Fisher’s LSD α = 0.05). Mean Ct values represent results from three biological replicates (n = 3). Error bars represent standard error of the mean. (TIF) [file pone.0319151.s004.tif]
