# Supplementary material for: Transcriptomic analysis reveals cloquintocet-mexyl-inducible genes in hexaploid wheat (Triticum aestivum L.)
Source: PLoS One. 2025 Feb 18;20(2):e0319151. doi: 10.1371/journal.pone.0319151 (PMC11835315; doi:10.1371/journal.pone.0319151)
Supplement: Attachment — Submitted filename: PONE-D-24-43694.docx [file pone.0319151.s011.docx]

**Manuscript Number:**  PONE-D-24-43694
**Manuscript Title:** Transcriptomic analysis reveals cloquintocet-mexyl-inducible genes in hexaploid wheat (*Triticum aestivum* L.)

**Reviewer:**

Dear editor,

The authors conducted a study to identify halauxifen acid (HA) detoxification genes using RNA-Seq. They compared untreated leaf tissue with leaf tissue treated with Cloquintocet-mexyl (CM). Through this analysis, they discovered the specific expression of CYP81A-5A and its homologues (CYP81A-5B and CYP81A-5D) in untreated leaf tissue, as well as in leaf tissue treated with CM and HM over time. While the findings of the study are innovative, further research is needed to fully support the claim that CYP81A-5A plays a role in herbicide detoxification. The results section should provide more detailed information on the number of differentially expressed genes (DEGs) and the gene ontology (GO) terms associated with transferase and oxidoreductase activity.

In summary, this study sheds light on potential HA detoxification genes, but additional studies are necessary to confirm the role of CYP81A-5A in this process.

**Abstract:**

Authors should provide a more detailed abstract regarding the number of reads that mapped to genes, as well as gene ontology analysis of differentially expressed genes.

**Material and methods:**

1. Please ensure that the names of all chemicals, the company name, and the percentage purity for each chemical are included in the Materials and Methods section of the manuscript.

2. The wording of line 98 should be formalize.

3. During the seedling stage, how was the plant irrigation performed before the treatment was administered?

4. When an abbreviation is used for the first time, it is not necessary to use the full name for subsequent mentions.

5. In line 120, why only one biological replicate per treatment per timepoint from each experimental replication was used for TaqMan RT-qPCR. It is advisable to include 3-5 biological replicates for each experimental condition in order to accurately measure experimental variation and establish the statistical significance of results. Additionally, it is recommended to conduct at least 3 technical replicates for each cDNA sample to assess assay variability and reduce the likelihood of pipetting errors.

**Results:**

1. Line 192, add the number of genes found through gene annotation that belong to the following categories: annotations, herbicide/xenobiotic metabolism, stress/defense response, amino acid metabolism, heavy metal binding, and encoded transcription factors. Specifically, indicate which genes were upregulated and which were downregulated in the category of herbicide/xenobiotic metabolism.

2. Line 195, The names of repressed genes need to be included.

3. In line 196, the author stated, "CM represses a few stress-related genes and likely triggers some level of oxidative stress, possibly to fine-tune safener-mediated transcriptional regulation." Author should be providing clarification on the number of genes that were suppressed.

4. The end of sentence of line 220 should be removed and relocated to the Materials and Methods.

5. In line 221, the author should specify the number of Gene Ontology (GO) terms associated with transferase activity, including both glutathione S-transferases (GST) and UDP-glucuronosyltransferases (UGTs), as well as oxidoreductase activity.

6. Line 236 should be shifted to M&M.

7. Which plant is involved with stress responses in UGT85As at line 247? Please include the name of the plant at the end of the sentence.

8. In line 257, the author stated that, "With the exception of TraesCS5A02G472300, these genes are located in tandem gene clusters encoding similar enzymes (Table S5)." Provide clarification on which specific genes are being referred to in this statement?

9. Line 271 should be relocated to the Methodology section of the report, as it is relevant to the methodology employed in the study, rather than the results obtained.

**Discussion:**

The discussion is too lengthy. It would be beneficial to eliminate redundant and overly informative sentences. For example, the sentence "Results of previous LC-MS [35] and phenotypic experiments [34] indicate that CYP81A-5D alone..." is repeated multiple times throughout the manuscript in the results, discussion, and conclusion sections. It is recommended to streamline the content and avoid unnecessary repetition for better clarity and conciseness.

**Conclusion:**

It is not customary to include references in the conclusion. Please remove them and instead, highlight the key findings of the study in the conclusion. Lines 415-420 should be shortened, and it is recommended that authors bold their key results for emphasis.

**Figures:**

- The red boxes in Supplementary Figures S1 and S2 were not sharpened.

- The supplementary figure S2 was not sharpened.

- In Supplementary Figure S4, there are errors in the significant letters for the 12- hours after treatment. Make the necessary corrections.
